# EEG Channel-Selection Method for Epileptic-Seizure Classification Based on Multi-Objective Optimization

**DOI:** 10.3389/fnins.2020.00593

**Published:** 2020-06-17

**Authors:** Luis Alfredo Moctezuma, Marta Molinas

**Affiliations:** Department of Engineering Cybernetics, Norwegian University of Science and Technology, Trondheim, Norway

**Keywords:** epilepsy, electroencephalogram (EEG), empirical mode decomposition (EMD), discrete wavelet transform (DWT), channel selection, multi-objective optimization, NSGA-II, NSGA-III

## Abstract

We present a multi-objective optimization method for electroencephalographic (EEG) channel selection based on the non-dominated sorting genetic algorithm (NSGA) for epileptic-seizure classification. We tested the method on EEG data of 24 patients from the CHB-MIT public dataset. The procedure starts by decomposing the EEG data from each channel into different frequency bands using the empirical mode decomposition (EMD) or the discrete wavelet transform (DWT), and then for each sub-band four features are extracted; two energy values and two fractal dimension values. The obtained feature vectors are then iteratively tested for solving two unconstrained objectives by NSGA-II or NSGA-III; to maximize classification accuracy and to reduce the number of EEG channels required for epileptic seizure classification. Our results have shown accuracies of up to 1.00 with only one EEG channel. Interestingly, when using all the EEG channels available, lower accuracies were achieved compared to the case when EEG channels were selected by NSGA-II or NSGA-III; i.e., in patient 19 we obtained an accuracy of 0.95 using all the channels and 0.975 using only two channels selected by NSGA-III. The results obtained are encouraging and it has been shown that it is possible to classify epileptic seizures using a few electrodes, which provide evidence for the future development of portable EEG seizure detection devices.

## 1. Introduction

Epilepsy is a group of neurological disorders, characterized by recurrent epileptic seizures, that affects approximately 1% of the world's population of all ages, both sexes, and all races and ethnic backgrounds (Mormann et al., 2006). It consists of widespread electrical discharges of a set of neurons inside the brain (Kale, 1997). Epileptic seizures are normally detected by continuous monitoring of electroencephalographic signals (EEG); the epileptiform can be categorized into ictal, interictal, and postictal periods. The identification of seizures by visual inspection can be time-consuming and lead to an incorrect interpretation of EEG signals, which can trigger under/over medication of patients (Engel, 1984). Suitable methods for detecting epileptic seizures could facilitate the rapid treatment of patients.

Current state-of-the-art efforts, such as that reported here, are attempting to improve the feature extraction stage for correct representation of the seizure and seizure-free periods. Several relevant studies using the same public dataset have been published, using various experimental setups. The study presented by Khan et al. (2012) used relative energy values and normalized variation coefficients from discrete wavelet transform (DWT) in the feature-extraction stage and then linear discriminant analysis (LDA) for classification. The method was evaluated on the data of five subjects, with 23, 24, or 26 channels, depending on the subject. In the classification process, they used approximately 80% of the data for training and the rest for testing, obtaining an accuracy of 0.91. Zabihi et al. (2015) later presented a method for feature extraction with even features from the intersection sequence of Poincaré section with phase space using LDA and naive Bayes classifiers. They used 23 channels, obtaining accuracies of 0.93 using 25% of the data for training and 0.94 using 50%.

The signal curve length of the time-domain EEG signal and the mode powers of the dynamic mode decomposition (DMD) have been used by Solaija et al. (2018) for feature extraction. They reported a sensitivity of 0.87 using approximately 50% of the data for training their models for epileptic-seizure classification.

We previously presented an approach using empirical mode decomposition (EMD) to decompose EEG signals into different intrinsic mode functions (IMF) and five features for each chosen IMF (Moctezuma and Molinas, 2019a). In that study, we presented results of an approach based on channel reduction using the backward-elimination algorithm, obtaining an average classification accuracy of 0.93 when five channels and 10-fold cross-validation were used.

Bhattacharyya and Pachori (2017) used a multivariate extension of empirical wavelet transform (EWT) to decompose the EEG signal into different oscillatory levels and compute three features for each level. The accuracies obtained ranged from 0.95 to 0.99 using five channels and various classifiers. This method selects the channel with the lowest standard deviation (SD) and then the remaining four channels with the highest mutual information (MI) with the previously chosen channel. Zhang et al. (2018) presented a method based on 24 feature types and the support vector machine (SVM) classifier. They used the TUH EEG Corpus (Obeid and Picone, 2016), the experiments were performed using 22 EEG channels and the accuracy obtained was 0.994.

There are some proposed methods using different values of entropy for feature extraction (Acharya et al., 2012), EMD for decomposing the EEG signals (Sharma and Pachori, 2015), using features based on Fourier-Bessel series expansion (Gupta and Pachori, 2019; Gupta et al., 2020), and with the energy from sub-bands extracted using the Taylor-Fourier filter bank (de la O Serna et al., 2020). The proposals used machine learning classifiers (Acharya et al., 2012; Sharma and Pachori, 2015; Gupta and Pachori, 2019; de la O Serna et al., 2020; Gupta et al., 2020), and neural networks (Sharma et al., 2020). However, these approaches have been tested using the Bonn university EEG database, which consist on a single channel and based on invasive seizure EEG signals (Andrzejak et al., 2001).

In addition to feature extraction and classifier design, a robust EEG channel selection procedure should reduce the computational cost to obtain results faster, decreasing possible over-fitting that comes from using all available channels. Recent efforts and advanced technology on dry EEG sensors have opened up new possibilities to develop new types of EEG systems (Fiedler et al., 2015; di Fronso et al., 2019). In this context, reducing the necessary number of EEG channels while maintaining or increasing the accuracy of machine-learning-based algorithms will be our targeted efforts toward low-cost portable devices for personal use.

Here, we analyze two methods for feature extraction, four classifiers with various parameters, and two channel selection methods to classify epileptic-seizure and seizure-free periods. We considered the process of selecting channels to be a multi-objective optimization problem, using the least possible number of EEG electrodes and obtaining the highest possible accuracy, we tested our approach on a well-known public dataset (Goldberger et al., 2000).

## 2. Materials and Methods

A laboratory setting and research-grade EEG equipment ensure a controlled environment and high-quality multiple-channel EEG recording. However, this approach is not suitable when considering a portable device for detecting epileptic seizures. This is because conventional EEG is challenged by high computational cost, high-density, non-portability of the equipment, and the use of inconvenient conductive gels. In addition, certain EEG channels may provide redundant information instead of helping to improve performance.

Epileptic seizure analysis using the complete EEG signals is not suitable for obtaining relevant information from the raw data and also for providing faster responses. Using feature extraction methods we can obtain relevant information not only in amplitude but also in frequency. With a set of features extracted, we can train a machine learning model that can reject new instances for prediction in real-time. The works presented in the literature suggest that using methods for feature extraction, is possible to improve the classifier's performance, especially decomposing the EEG signals into different frequency bands, using EMD or DWT (Khan et al., 2012; Sharma and Pachori, 2015; Moctezuma and Molinas, 2019a). The selection of the machine learning method that works better for epileptic seizure classification is also relevant and it has been studied in the literature, however, depending on the feature extraction methods, the classifier's performance may vary. Following our previous findings, here we compare four different classifiers, as it is explained in section 2.3.

We performed the experiments for this study on the NTNU IDUN computing cluster Själander et al., 2019. The cluster has more than 70 nodes and 90 GPGPUs. Each node contains two Intel Xeon cores and at least 128 GB of main memory and is connected to an Infiniband network. Half of the nodes are equipped with two or more Nvidia Tesla P100 or V100 GPGPUs. Idun's storage is provided by two storage arrays and a Luster parallel distributed file system.

### 2.1. Patients and EEG Recording

For the comparison of any proposed method and its performance, the use of free and public EEG-signals datasets is important. Most of the proposed methods in the state-of-the-art are tested on datasets from the PhysioNet (Goldberger et al., 2000) and EPILEPSIAE (Dourado et al., 2009) projects, and from the TUH EEG Corpus (Obeid and Picone, 2016), where some of the datasets consist of private repositories or access is limited.

The EEG recordings used were obtained from pediatric patients with intractable seizures who were monitored for several days at the Boston Children's Hospital following the withdrawal of anti-seizure medication to characterize their seizures and assess their candidacy for surgical intervention. The dataset used comes from the PhysioNet project and is partially described in Goldberger et al. (2000) and Shoeb (2009). The dataset consists of bipolar EEG signals from 24 patients that were recorded using 22 channels (FP1-F7, F7-T7, T7-P7, P7-O1, FP1-F3, F3-C3, C3-P3, P3-O1, FP2-F4, F4-C4, C4-P4, P4-O2, FP2-F8, F8-T8, P8-O2, FZ-CZ, CZ-PZ, P7-T7, T7-FT9, FT9-FT10, FT10-T8, and T8-P8), with a sampling rate of 256 Hz using the 10-20 international system. It should be noted that channels FT9 and FT10 are not part of the 10-20 international system, for illustrative purposes, we use the 10-10 system when necessary.

The EEG data for each epileptic seizure and epileptic-free period is of 6 s and there are 80 instances on average for each class for each patient. The EEG signals were down-sampled to 128 Hz as our previous research has been shown that the results did not differ using 256 or 128 Hz, however, the process for decomposing the EEG signals into different sub-bands is faster with 128 Hz (Moctezuma and Molinas, 2019a). More details can be found in Goldberger et al. (2000), Shoeb (2009), and Moctezuma and Molinas (2019a).

### 2.2. Feature Extraction

#### 2.2.1. Empirical Mode Decomposition

EMD is a decomposition method that can deal with non-linear and non-stationary signals and is based on the local characteristic time scale of the data, is adaptive and offers physical meaning. EMD decomposes an EEG signal into a finite set of oscillatory components, known as IMFs, by applying the sifting process, as it is shown in **Algorithm 1** (Huang et al., 1998). During the sifting process, some redundant IMFs with shape and frequency content different from those of the original signal may appear. These inappropriate IMFs show maximum Minkowski (Euclidean) distances with respect to the original signal (Boutana et al., 2010). We tested using different numbers of IMFs but used the closest two IMFs according to the Minkowski / Euclidean distance because they showed the same performance as that of using more. We characterized each selected IMF and reduced the data dimension by extracting four features, which are described in section 2.2.3.

**Algorithm 1:** The sifting process for a signal *x*(*t*).

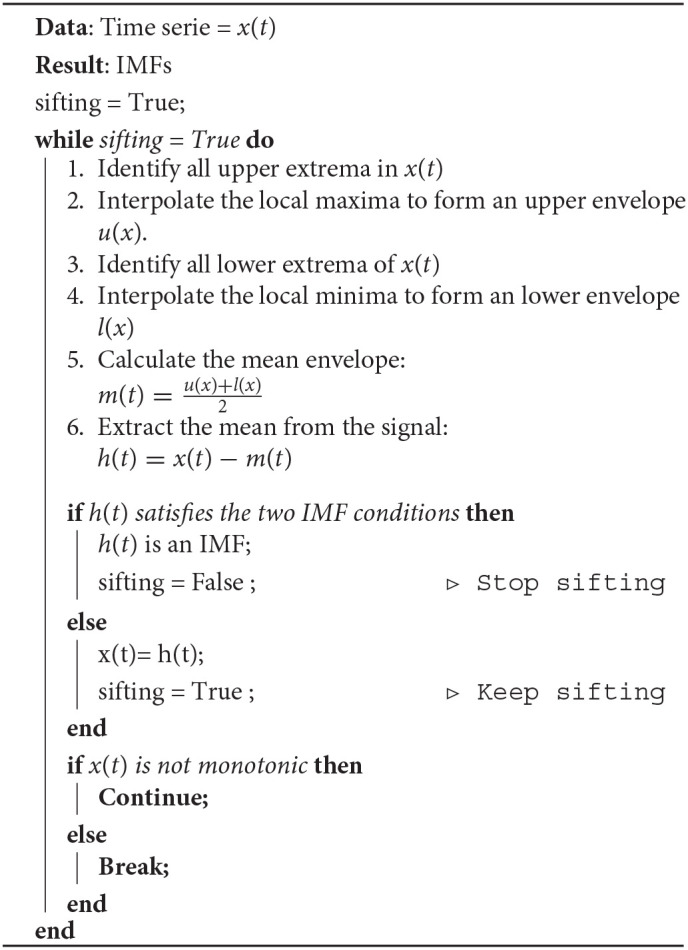

#### 2.2.2. Discrete Wavelet Transform

DWT is a decomposition method that can deal with non-stationary signals, and decomposes the EEG signal into different frequency sub-bands, but does not offer a physical interpretation for the components. When using DWT, it is necessary to specify the mother function and the levels of decomposition. The mother (prototype) wavelet (functions) is scaled or dilated to decompose a signal in the time-domain into shifted and scaled versions of a base wavelet. Its outputs provide in the first level a high-frequency part known as detail coefficients (D1), and a low-frequency part, known as approximation coefficients (A1). Then, the low-frequency part is used as input for generating another decomposition level until the predefined number of levels of decomposition is reached. In short, the wavelet decomposition of a signal *S* in the *j* decomposition level has the structure [*A*_*j*_, *D*_*j*_, *D*_*j*−1_, ..., *D*_1_], it should be noted that at every level, half of the samples can be removed according to the Nyquist theorem (Mallat, 1989).

Here, we use the mother function bi-orthogonal 2.2 with four levels of decomposition, based on the results obtained from previous studies (Moctezuma, 2017; Moctezuma and Molinas, 2019b; Moctezuma et al., 2019). We obtained most of the brain rhythms (32–64, 16–32, 8–16, 4–8, and 0–4 Hz) using these decomposed components.

#### 2.2.3. Features

Both, EMD and DWT sub-bands were used as inputs for a method that extracts four features for each sub-band. The method for feature extraction consisted of computing two energy values and two fractal dimension values from both the EMD (Moctezuma and Molinas, 2018, 2020) and DWT sub-bands (Moctezuma and Molinas, 2019b). This set of features are introduced in Moctezuma and Molinas (2018), Moctezuma and Molinas (2019a), and Moctezuma and Molinas (2020) and described below.

- Instantaneous Energy: gives the energy distribution in log base ten for each band (Didiot et al., 2010):

(1) $$\begin{array}{r} f_{j}=log_{10}\left(\frac{1}{N_{j}}\overset{N_{j}}{\underset{r=1}{\sum }}{\left(w_{j}\left(r\right)\right)}^{2}\right) \end{array}$$

- Teager Energy: This log base ten energy operator reflects variations in both amplitude and frequency of the signal (Jabloun and Cetin, 1999; Didiot et al., 2010):

(2) $$\begin{array}{r} f_{j}=log_{10}\left(\frac{1}{N_{j}}\overset{N_{j}-1}{\underset{r=1}{\sum }}|{\left(w_{j}\left(r\right)\right)}^{2}-w_{j}\left(r-1\right)*w_{j}\left(r+1\right)|\right) \end{array}$$

- Higuchi Fractal Dimension: The algorithm approximates the mean length of the curve using segments of *k* samples and estimates the dimension of a time-varying signal directly in the time domain (Higuchi, 1988). Considered a finite set of observations taken at a regular interval: *X*(1), *X*(2), *X*(3), .., *X*(*N*). From this series, a new one $X_{k}^{m}$ must be constructed,

(3) $$X_{k}^{m}:X(m),X(m+k),X(m+2k),..,X\left(m+\left(\frac{N-m}{k}\right)k\right)$$

Where *m* = 1, 2, .., *k*, *m* indicate the initial time and *k* the interval time. Then, the length of the curve associated to each time series $X_{k}^{m}$ can be computed as follow:

(4) $$L_{m}(k)=\frac{1}{k}\left(\sum_{i=1}^{\frac{N-m}{k}}\left(X(m+ik)-X\left(m+(i-1)k\right)\right)\right)\left(\frac{N-1}{\left(\frac{N-m}{k}\right)k}\right)$$

Higuchi takes the mean length of the curve for each *k*, as the average value of *L*_*m*_(*k*), for *m* = 1, 2, ..., *k* and *k* = 1, 2, ..., *k*_*max*_, that it is calculated as:

(5) $$\begin{array}{r} L\left(k\right)=\frac{1}{k}\overset{k}{\underset{m-1}{\sum }}\left(L_{m}\left(k\right)\right) \end{array}$$

- Petrosian Fractal Dimension: can be used to provide a fast computation of the fractal dimension of a signal by translating the series into a binary sequence (Petrosian, 1995).

(6) $$\begin{array}{r} FD_{Petrosian}=\frac{\log_{10}n}{\log_{10}n+\log_{10}\left(\frac{n}{n+0.4N_{\nabla }}\right)} \end{array}$$

Where *n* is the length of the sequence and *N*_∇_ is the number of sign changes in the binary sequence.

For each EEG channel, we obtained a number of features, eight for EMD and 20 for DWT, that were concatenated to represent all epileptic-seizure or seizure-free periods. Repeating this process with all the instances allowed us to obtain a balanced dataset that was used as input for the classifiers.

### 2.3. Classification

Deep learning algorithms have been shown to be a success in image processing and other fields, but when using EEG data they have not shown convincing and consistent improvements over the most advanced methods to date (Lotte et al., 2018). Additionally, its performance depends on the use of a large number of instances, something that is not common when using EEG data. In our case, we used some classifiers that have been shown to be effective with little training data (Tsoumakas and Katakis, 2007; Akram et al., 2015; Steyrl et al., 2016; Zhang et al., 2017; Lotte et al., 2018), and it has been presented in our previous research that such classifiers present similar results depending on the number of channels, the number of instances, and the method for feature extraction used (Moctezuma and Molinas, 2019a,b, 2020; Moctezuma et al., 2019).

The first classifier used was the well-known SVM, as it provides a global solution and the classification complexity does not depend on the feature dimension Joachims (1998). For SVM, the kernels tested are sigmoid, linear, and radial basis functions (RBFs). The second classifier was the k-nearest neighbors (KNN) classifier, with *1–9* neighbors. Random forest (RF) was also tested using different tree depths, which can be *2–5*. Finally, the naive Bayes (NB) classifier was also tested to analyze its performance for this task.

For classification, we tested all four classifiers and only that which showed the highest accuracy was retained (**Figure 2**), meaning that a different classifier may be used for each subset of channels. The implementation of each classifier internally selects the best parameters by testing the set of possible parameters in each case, for instance, KNN was tested with 1–9 neighbors, but the number of neighbors used in the classifier was the one with the highest accuracy. We use 10-fold cross-validation to evaluate the performance of each classifier. It should be noted that 9-fold cross-validation was applied when required, depending on the lowest number of trials per class in the patients, i.e., in the case of subject 16, according to the information described in Goldberger et al. (2000), Shoeb (2009), and Moctezuma and Molinas (2019a).

### 2.4. EEG Channel Selection

This process is essential for decreasing the computational cost, making it possible to obtain the results more quickly and consider portable low-cost headsets. It allows focusing on the channels containing the most information, thus maintaining or even increasing classification accuracy. First, we briefly explain a method for channel reduction and then present two multi-objective optimization algorithms.

#### 2.4.1. Backward-Elimination Algorithm

This well-known *greedy* algorithm, has been used for feature subset selection (Narendra and Fukunaga, 1977; Foroutan and Sklansky, 1987; Yang and Honavar, 1998) and channel reduction using EEG signals (Moctezuma and Molinas, 2019a).

This algorithm begins by testing all possible combinations by removing one channel at a time. Feature extraction and classification is performed for each subset and that with the highest accuracy is used in the next iteration to eliminate another channel until there is one left. For the dataset used here, the classification process was performed $\frac{22*23}{2}=253$ times for each patient.

This provides a general indication of the channels containing less information because it provides an optimal solution at each step, but does not consider the complex iterations of the channels that could affect classification accuracy.

#### 2.4.2. NSGA-II

The genetic algorithms (GAs), which mimic Darwinian evolution, are normally used to solve complex optimization and search problems (Chugh et al., 2019). The population for GAs is comprised of a set of candidate solutions, each with chromosomes than can be mutated and altered.

In a multi-objective optimization problem, there is a set of solutions that is superior to the others in the search space when all the objectives are considered, but inferior to the other solutions for one or more objectives. Such solutions are known as Pareto-optimal solutions or *non-dominated solutions* and the rest as dominated solutions. The non-dominated sorting ranking selection method is used to emphasize good candidates and a niche method is used to maintain stable sub-populations of good points. The non-dominated sorting genetic algorithms (NSGA) were created based on this concept (Srinivas and Deb, 1994).

The first version of NSGA showed problems related to the computational complexity, non-elitism approach, and need to specify a sharing parameter to ensure diversity in a population. NSGA-II reduced the computational cost from *O*(*MN*^3^) to *O*(*MN*^2^), where *M* is the number of objectives and *N* the population size. Additionally, the elitism approach was introduced by comparing the current population with the previously found best non-dominated solutions (Deb et al., 2002). NSGA-II elitism does not require the setting of any new parameters other than the normal genetic algorithm parameters, such as population size, termination parameter, and crossover and mutation probabilities.

**Figure 1:**
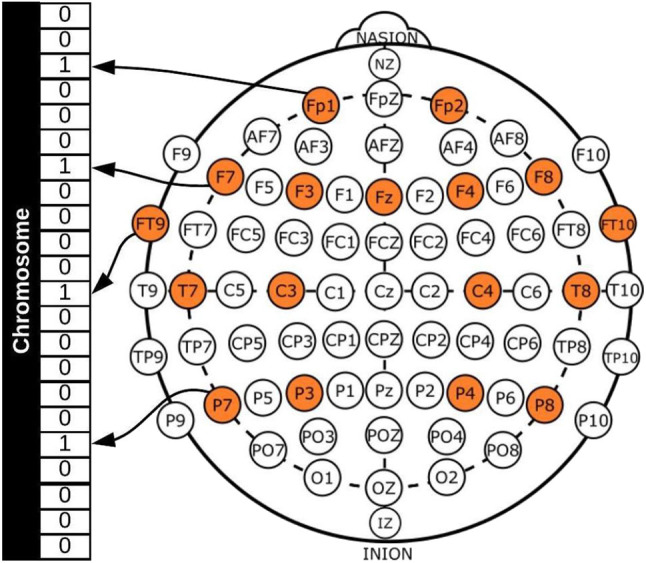
Example of channel representation in a chromosome for a GA.

In general, a GA requires a genetic representation of the solution domain and a fitness function to evaluate the solutions domain, which in this case, was an array representing each channel (see Figure 1) and the fitness function for the two-objective optimization problem was defined as [*Acc, No*], where *Acc* was the classification accuracy obtained with the chromosome and *No* the number of EEG channels used.

Figure 1 shows a binary representation for creating the chromosomes, with each gene representing a channel, 1 if the channel will be used for the classification process and 0 if not. All possible channels that can be used are colored and this represents the search space, which is 22, as already mentioned. It should be noted that channels FP1-F7, FP1-F3, T7-P7, T7-FT9, P7-T7, P7-O1, FP2-F4, and FP2-F8 were considered to be different, as the references for the channels are different and the dataset provides the EEG signals for each one separately.

#### 2.4.3. NSGA-III

This method follows the NSGA-II framework using a set of supplied or predefined reference points that emphasizes population members that are non-dominated, yet close to the supplied set (Deb and Jain, 2013; Jain and Deb, 2013). It has shown its efficiency in solving two-objective to 15-objective optimization problems (Deb and Jain, 2013).

The predefined set of reference points are used to ensure diversity in the obtained solutions and can be predefined in a structured manner or defined in the problem to be optimized by the user. Here, we used a systematic approach for creating the reference points presented by Das and Dennis (1998), as in Jain and Deb (2013). This approach places points on a normalized hyper-plane that is equally inclined to all objective axes and has an interception of one on each axis. For example, in a three-objective optimization problem, the reference points are created on a triangle with apexes at (1, 0, 0), (0, 1, 0), and (0, 0, 1).

### 2.5. Problem and System Definition for NSGA-II and NSGA-III

All the best solutions found in the optimization process for epileptic-seizure classification were analyzed. There are some applications using EEG signals in which the automatic selection of the best solution may be important, especially for cross-subject analysis. Here, however, it was important to analyze all the results for each patient individually. With this assumption, a possible low-cost EEG headset designer can consider whether it is better to sacrifice accuracy or the number of EEG channels, depending on how easy or difficult it is to detect epileptic seizures for a given individual.

**Figure 2:**
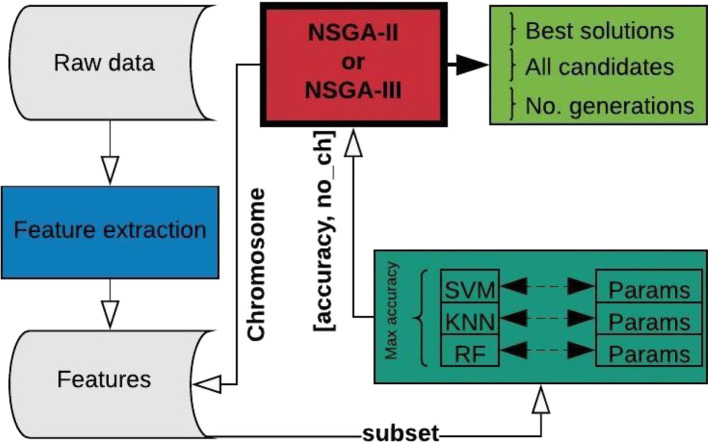
Complete process for EEG channel selection using NSGA-II or NSGA-III.

The problem to be optimized is defined by two unconstrained objectives: first, to maximize accuracy and second, to decrease the number of channels used for epileptic seizure classification. The termination criterion for the optimization process is defined by the objective space tolerance, which is defined as 0.0001. This criterion is calculated every 5th generation and if not achieved, the process stops after a maximum of 500 generations. Figure 2 shows the complete process, which consists of three main stages: feature extraction, classification, and optimization.

The process starts using the raw EEG signals of one patient at a time, from which feature extraction is performed and the results organized and stored for iterative use (Figure 2). From this point on, the main process is handled by the NSGA, which starts creating all the possible candidates (chromosomes) for each population, obtaining the corresponding subset of features for the channels represented as 1 in the chromosome, and evaluating the subset with four different classifiers, with different parameters for each. The best accuracy obtained and the number of EEG channels used is returned to the NSGA to evaluate each chromosome in the current population. The process is repeated, creating different populations by the NSGA until the termination criterion is reached.

In summary, the chromosome has 22 genes, each representing an EEG channel. Each population size in each iteration is defined as 20, which was selected experimentally. Four classifiers were tested for each possible solution, but only the highest accuracy was retained and the corresponding classifier used was stored for analytical purposes.

One of the objectives of this study was to compare our approach with the state-of-the-art and present easily reproducible results. We thus used free public tools for creating the code. Implementation of the classifiers is based on the scikit-learn python library presented by Pedregosa et al. (2011). NSGA-II and NSGA-II are based on pymoo presented by Blank and Deb (2020).

## 3. Results

We performed classification experiments using the characterized EEG signals for each patient separately, while reducing or selecting the EEG channels for creating models to detect epileptic seizures. For each patient, a carefully balanced dataset was created using epileptic-seizure and seizure-free segments of 6-s.

### 3.1. Epileptic-Seizure Classification Using EMD

For this experiment, we used EMD-based feature extraction, the greedy algorithm for channel reduction, and both NSGA-II and NSGA-III for channel selection. The process described in 2.5 was repeated for each patient using the above techniques.

**Figure 3:**
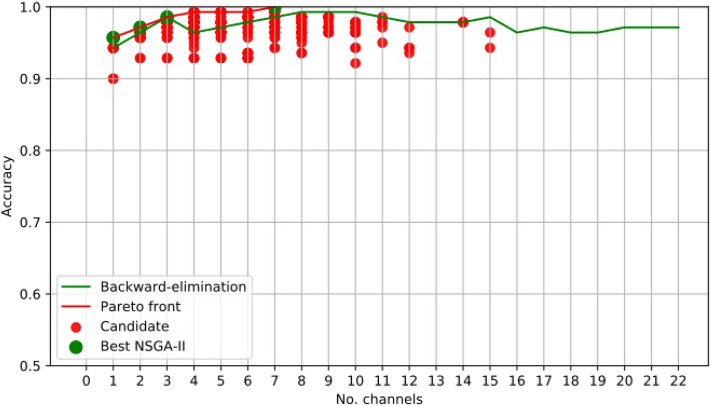
EEG Channel Selection for epileptic seizure classification of patient 1, using EMD-based features. Comparison using NSGA-II and the backward-elimination algorithm.

For illustrative purposes, Figure 3 presents the results obtained using NSGA-II for epileptic-seizure classification of patient 1.

Figure 3 clearly shows that NSGA-II managed to cope with both objectives, whereas, although the backward-elimination algorithm sometimes showed higher accuracy when using a high number of channels, the opposite was true when using a lower number of channels.

**Figure 4:**
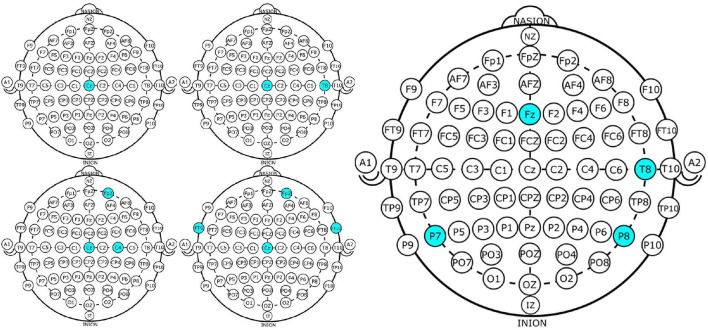
Four EEG Channel subsets selected by NSGA-II **(left)** and backward-elimination **(right)** for epileptic-seizure classification in patient 1.

In this case, the best results obtained using NSGA-II consisted of four subsets of channels, which did not necessarily overlap. This is because each chromosome was almost independent and may have come from different parents. The illustrative example presented in Figure 4 shows the subsets of channels used for obtaining the highest accuracy.

Channel *Cz* was selected in the first four subsets shown using the NSGA-II method, but not when backward-elimination was used. The accuracy obtained by backward-elimination was notably lower than when NSGA-II was used (0.964 and 0.993, respectively) (Figure 3), which shows the feasibility of the method, as well as the importance of a robust method for channel selection.

Tables 1, 2 shows the accuracies obtained using each of the methods on data from all of the patients. Most of the best results were obtained when 10 channels were reduced to one (Figure 3). The tables show only the results for channels 1–10 for all patients, but the experiment was carried out with all channels. As we used an automatic termination criterion, the number of generations for each patient was different and is shown in the tables. Supplementary Material  provides accuracies, specificities, and sensitivities for the first 4 EEG channels of Tables 1, 2.

**Table 1:** Accuracies obtained using EMD for feature extraction with NSGA-II and NSGA-III for EEG channel selection (Subjects 1–12).

**Id**	**Method**	**Gen**.	**No. channels**									
			**1**	**2**	**3**	**4**	**5**	**6**	**7**	**8**	**9**	**10**
1	B-E	–	0.943	0.964	0.986	0.964	0.971	0.979	0.986	0.993	0.993	0.993
	NSGA-II	30	0.979	0.979	0.986	0.993						
	NSGA-III	60	0.964	0.979					1.000			
2	B-E	–	0.815	0.899	0.921	0.921	0.961	0.976	0.969	0.985	0.985	0.985
	NSGA-II	40	0.866	0.921								
	NSGA-III	40	0.866									
3	B-E	–	0.796	0.888	0.912	0.920	0.960	0.976	0.969	0.985	0.985	0.985
	NSGA-II	30	0.911	0.943	0.958	0.975		0.976	0.975			
	NSGA-III	70	0.876	0.927	0.951	0.975	0.976					
4	B-E	–	0.832	0.940	0.948	0.977	0.976	0.985	0.977	0.986	0.986	0.986
	NSGA-II	40	0.914	0.946	0.955	0.977	0.992					
	NSGA-III	40	0.897	0.955	0.963			1.000				
5	B-E	–	0.972	0.978	0.995	1.000	1.000	1.000	1.000	1.000	1.000	1.000
	NSGA-II	30	0.974	0.995	1.000							
	NSGA-III	30	0.970	0.995								
6	B-E	–	0.975	1.000	0.975	1.000	1.000	0.975	1.000	1.000	1.000	1.000
	NSGA-II	30	1.000	1.000								
	NSGA-III	30	1.000	1.000								
7	B-E	–	0.962	0.962	0.963	0.992	0.992	0.992	0.992	0.992	0.992	0.992
	NSGA-II	50	0.962	0.972	0.982	1.000						
	NSGA-III	60	0.962	0.972		1.000						
8	B-E	–	0.884	0.884	0.877	0.877	0.874	0.877	0.865	0.884	0.874	0.890
	NSGA-II	40	0.884	0.890	0.890	0.890						
	NSGA-III	50	0.884	0.884								
9	B-E	–	1.000	1.000	1.000	1.000	1.000	1.000	1.000	1.000	1.000	1.000
	NSGA-II	30	1.000									
	NSGA-III	30	1.000									
10	B-E	–	0.993	0.993	0.993	1.000	1.000	1.000	1.000	1.000	1.000	1.000
	NSGA-II	30	0.993	1.000								
	NSGA-III	40	0.993	1.000								
11	B-E	–	0.996	0.996	0.996	0.992	0.996	0.992	0.992	0.992	0.992	0.996
	NSGA-II	30	0.996	0.996								
	NSGA-III	40	0.996	0.996								
12	B-E	–	0.899	0.892	0.918	0.911	0.921	0.925	0.925	0.929	0.922	0.925
	NSGA-II	50	0.899	0.908	0.919	0.928	0.932	0.941				
	NSGA-III	70	0.899	0.912				0.942				

*Gray values are highlighted the higher accuracy between the methods for the channels*.

**Table 2:** Accuracies obtained using EMD for feature extraction with NSGA-II and NSGA-III for EEG channel selection (Subjects 13–24).

**Id**	**Method**	**Gen**.	**No. channels**									
			**1**	**2**	**3**	**4**	**5**	**6**	**7**	**8**	**9**	**10**
13	B-E	–	0.775	0.777	0.775	0.806	0.788	0.726	0.749	0.782	0.782	0.733
	NSGA-II	40	0.775	0.777	0.798	0.806			0.813			
	NSGA-III	40	0.775	0.777			0.813					
14	B-E	–	0.925	0.933	0.942	0.942	0.942	0.967	0.967	0.983	0.983	0.983
	NSGA-II	40	0.933	0.967	0.983	0.983						
	NSGA-III	40	0.933	0.942	0.983							
15	B-E	–	0.971	0.969	0.978	0.981	0.985	0.986	0.986	0.988	0.988	0.988
	NSGA-II	40	0.981	0.981	0.988	0.988						
	NSGA-III	40	0.981	0.985	0.988							
16	B-E	–	0.900	0.900	0.900	0.900	0.900	0.900	0.900	0.900	0.900	0.800
	NSGA-II	70	0.900	0.900								
	NSGA-III	70	0.900	0.900								
17	B-E	–	0.940	0.980	0.980	0.990	1.000	1.000	1.000	1.000	1.000	1.000
	NSGA-II	30	0.980	0.990	1.000							
	NSGA-III	40	0.980		1.000							
18	B-E	–	0.790	0.852	0.832	0.862	0.853	0.882	0.892	0.910	0.900	0.900
	NSGA-II	70	0.803	0.852	0.870	0.900		0.910	0.920			
	NSGA-III	40	0.783	0.852	0.862	0.880	0.890	0.892				
19	B-E	–	0.913	0.908	0.925	0.925	0.950	0.963	0.975	0.975	0.988	0.988
	NSGA-II	30	0.921	0.946	0.950	0.963	0.975	0.988	1.000			
	NSGA-III	60	0.913	0.975				1.000				
20	B-E	–	0.948	0.970	0.957	0.957	0.970	0.980	0.990	0.990	0.968	0.980
	NSGA-II	30	0.980		0.990							
	NSGA-III	50	0.980		0.990							
21	B-E	–	0.879	0.933	0.888	0.888	0.908	0.938	0.904	0.942	0.933	0.908
	NSGA-II	30	0.888	0.950	0.954	0.967	0.970	0.983				
	NSGA-III	50	0.888	0.942	0.954	0.983						
22	B-E	–	0.971	0.971	0.983	0.983	0.983	0.983	0.983	0.983	0.983	0.983
	NSGA-II	50	0.983		0.983							
	NSGA-III	60	0.983									
23	B-E	–	0.938	0.940	0.938	0.955	0.962	0.955	0.962	0.962	0.962	0.962
	NSGA-II	40	0.938	0.948	0.962							
	NSGA-III	40	0.938	0.946				0.970				
24	B-E	–	0.975	0.975	0.992	0.992	0.992	0.992	0.992	0.992	0.992	0.992
	NSGA-II	40	0.975	0.992	0.992	1.000						
	NSGA-III	40	0.992			1.000						

*Gray values are highlighted the higher accuracy between the methods for the channels*.

The results highlighted in gray are those for which the accuracy obtained was higher than when using backward-elimination. The average number of generations was 39 ± 12 for NSGA-II and 47 ± 13 for NSGA-III.

Patient 13 appears to be a possible special case, as similar accuracies were obtained with all methods. NSGA-II showed the highest accuracy when using three channels and NSGA-III when using five, reaching 0.813. The addition of more channels to detect epileptic seizures resulted in fluctuations in the accuracy but it did not increase.

**Figure 5:**
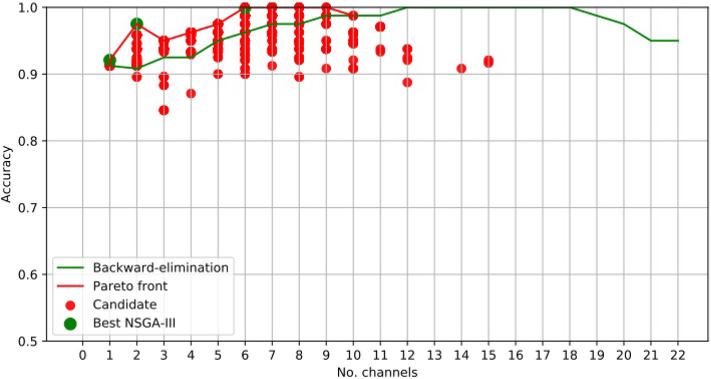
EEG Channel selection for epileptic-seizure classification of patient 19, using EMD-based features. Comparison using NSGA-III and the backward-elimination algorithm.

Table 2 shows a number of empty cells when using NSGA-II and NSGA-III, meaning that the accuracy obtained was not part of the best solutions. This is best illustrated for the results obtained for patient 19 using the NSGA-III method (Figure 5). This case shows a clear example of how the method works, as the accuracy obtained using two channels was 0.975 but the addition of more channels only decreased the accuracy, except for the use of six channels. This is related to the small amount of information provided by the aggregate channels.

**Figure 6:**
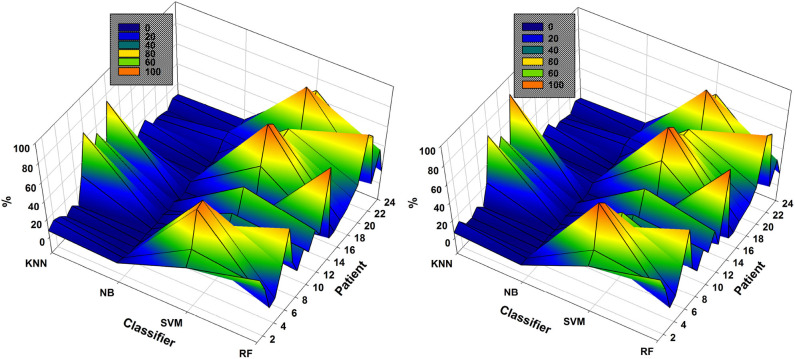
Comparison of the most used classifiers by NSGA-II **(left)** and NSGA-III **(right)** in the 24 patients with EMD-based feature extraction.

As mentioned previously, the classifier used each time is that resulting in the highest accuracy using the subsets of EEG channels. The NSGA-based algorithms were clearly able to handle the complete process and the classifiers most used to obtain the highest accuracies are presented in Figure 6. The results show the percentage of use of each classifier for each patient. We use the percentage of use, as the number of generations for each patient was different, depending on the method used for feature extraction, as well as for EEG channel selection. For example, in the case of NSGA-II for patient 1, the most highly used classifier was RF, which was used 54.59% of the time, then SVM with 33.72%, k-NN with 7.35%, and NB with 4.34%.

SVM and RF were the most highly used classifiers to obtain the highest accuracy in all iterations of NSGA-II and NSGA-III (Figure 6). On the other hand, NB was used in all iterations but only returned the highest accuracy a few times. In general, RF was used 32.8% ± 24.2 of the time for all patients, SVM 47.0% ± 27.9, NB 3.1% ± 4.2, and KNN 17.1% ± 20.5. For NSGA-III, the RF classifier was used 32.0% ± 25.1 of the time, SVM 48.8% ± 28.6, NB 2.8% ± 3.6, and KNN 16.4% ± 21.7.

The analysis of the most highly used classifier in all generations and each chromosome is important because it allows discarding the use of some to decrease the computational cost and also because it shows that the classifier necessary to obtain the highest accuracy may differ, depending on the patient and the EEG channel subsets used.

### 3.2. DWT-Based Epileptic-Seizure Classification

We repeated the experiment but used DWT for feature extraction to extract the sub bands and then computed the four features, as described above. The experiments were repeated using NSGA-II and NSGA-III for the 24 patients. Additionally the accuracies obtained were also compared to those obtained using the backward-elimination algorithm. The results are summarized in Tables 3, 4, and Supplementary Material provides accuracies, specificities, and sensitivities for the first 4 EEG channels.

**Table 3:** Accuracies obtained using DWT for feature extraction with NSGA-II and NSGA-III for EEG channel selection (subjects 1–12).

**Id**	**Method**	**Gen**.	**No. channels**									
			**1**	**2**	**3**	**4**	**5**	**6**	**7**	**8**	**9**	**10**
1	B-E	–	0.950	0.993	0.993	0.993	1.000	0.993	0.993	0.993	1.000	1.000
	NSGA-II	30	0.986	1.000								
	NSGA-III	50	0.986		1.000							
2	B-E	–	0.983	0.992	0.992	1.000	1.000	1.000	1.000	1.000	1.000	1.000
	NSGA-II	30	0.992	0.992	1.000							
	NSGA-III	30	0.992	0.992		1.000						
3	B-E	–	0.983	0.985	0.992	1.000	1.000	1.000	1.000	1.000	1.000	1.000
	NSGA-II	40	0.983	0.992	1.000							
	NSGA-III	30	0.983		1.000							
4	B-E	–	0.952	0.966	0.975	0.983	0.976	0.983	0.983	0.983	0.976	0.983
	NSGA-II	30	1.00									
	NSGA-III	30	1.00									
5	B-E	–	0.995	1.000	1.000	1.000	1.000	1.000	1.000	1.000	1.000	1.000
	NSGA-II	30	1.000									
	NSGA-III	30	1.000									
6	B-E	–	0.975	0.950	0.950	0.950	0.950	0.950	0.950	0.950	0.900	1.000
	NSGA-II	50	0.975	0.975	0.975							
	NSGA-III	60	0.975	0.975				1.000				
7	B-E	–	0.962	0.972	0.980	0.980	0.980	0.980	0.980	0.980	0.980	0.980
	NSGA-II	40	0.980	0.982	1.000							
	NSGA-III	50	0.980		1.000							
8	B-E	–	0.914	0.903	0.917	0.904	0.894	0.884	0.894	0.890	0.890	0.894
	NSGA-II	50	0.917	0.917								
	NSGA-III	50	0.971		0.917							
9	B-E	–	1.000	1.000	1.000	1.000	1.000	1.000	1.000	1.000	1.000	1.000
	NSGA-II	30	1.000	1.000								
	NSGA-III	30	1.000									
10	B-E	–	1.000	1.000	1.000	1.000	1.000	1.000	1.000	1.000	1.000	1.000
	NSGA-II	30	1.000									
	NSGA-III	30	1.000	1.000								
11	B-E	–	1.000	1.000	1.000	1.000	0.996	0.996	0.996	1.000	0.996	1.000
	NSGA-II	30	1.000									
	NSGA-III	30	1.000									
12	B-E	–	0.899	0.932	0.942	0.942	0.949	0.935	0.942	0.945	0.952	0.945
	NSGA-II	30	0.911	0.948	0.948	0.952						
	NSGA-III	40	0.911						0.952			

*Gray values are highlighted the higher accuracy between the methods for the channels*.

**Table 4:** Accuracies obtained using DWT for feature extraction with NSGA-II and NSGA-III for EEG channel selection (subjects 13–24).

**Id**	**Method**	**Gen**.	**No. channels**									
			**1**	**2**	**3**	**4**	**5**	**6**	**7**	**8**	**9**	**10**
13	B-E	–	0.822	0.827	0.793	0.827	0.795	0.798	0.776	0.798	0.776	0.827
	NSGA-II	40	0.820	0.849		0.855		0.864				
	NSGA-III	50	0.820			0.850						
14	B-E	–	0.950	0.967	0.983	0.983	0.983	1.000	1.000	1.000	1.000	1.000
	NSGA-II	40	0.967	0.983	0.995							
	NSGA-III	40	0.967	0.983		1.000						
15	B-E	–	0.978	0.985	0.981	0.986	0.986	0.988	0.994	0.995	0.998	0.997
	NSGA-II	40	0.978	0.994	1.000							
	NSGA-III	50	0.978	0.994	0.998		1.000					
16	B-E	–	0.800	1.000	1.000	1.000	1.000	1.000	1.000	1.000	1.000	1.000
	NSGA-II	30	1.000									
	NSGA-III	30	1.000									
17	B-E	–	0.930	1.000	1.000	1.000	1.000	1.000	1.000	1.000	1.000	1.000
	NSGA-II	30	1.000									
	NSGA-III	30	1.000									
18	B-E	–	0.862	0.862	0.912	0.922	0.922	0.922	0.940	0.952	0.932	0.952
	NSGA-II	40	0.890	0.913	0.950			0.952				
	NSGA-III	50	0.862	0.913			0.952					
19	B-E	–	0.987	1.000	0.987	1.000	1.000	1.000	1.000	1.000	1.000	1.000
	NSGA-II	30	0.988	1.000								
	NSGA-III	30	0.988	1.000								
20	B-E	–	1.000	1.000	1.000	1.000	1.000	0.990	0.990	0.990	1.000	0.990
	NSGA-II	30	1.000									
	NSGA-III	30	1.000									
21	B-E	–	0.921	0.950	0.938	0.967	0.983	0.966	0.966	0.966	0.966	0.966
	NSGA-II	40	0.925	0.950	0.971	0.983						
	NSGA-III	50	0.933	0.950			0.983					
22	B-E	–	0.983	0.983	0.983	0.983	0.983	0.983	0.983	0.983	0.983	0.983
	NSGA-II	40	0.995	0.998	1.000							
	NSGA-III	50	0.995	0.995								
23	B-E	–	0.938	0.946	0.953	0.961	0.961	0.962	0.955	0.962	0.969	0.969
	NSGA-II	40	0.939	0.961	0.969	0.970	0.970	0.977				
	NSGA-III	60	0.939	0.961					0.977			
24	B-E	–	0.975	0.975	0.975	0.975	0.975	0.983	0.975	0.983	0.975	0.983
	NSGA-II	40	0.985	0.992	1.000							
	NSGA-III	40	0.985	0.988		1.000						

*Gray values are highlighted the higher accuracy between the methods for the channels*.

The results in Tables 3, 4 show that an average of 36 ± 7 generations was required for NSGA-II and 41 ± 11 for NSGA-III. In general, the use of DWT for feature extraction resulted in more rapid EEG channel selection and better accuracy.

**Figure 7:**
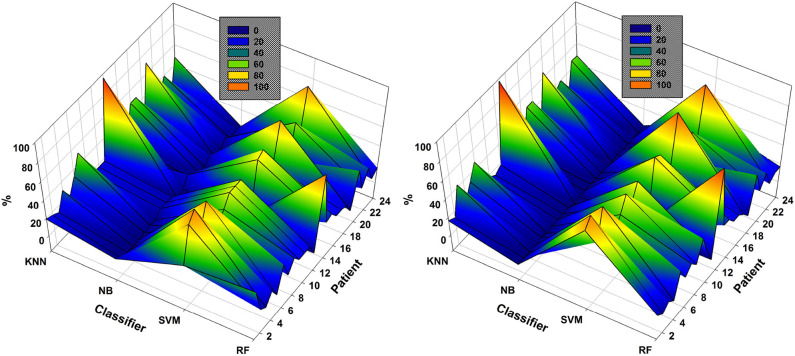
Comparison of the most-used classifiers by NSGA-II **(left)** and NSGA-III **(right)** for the 24 patients with DWT-based feature extraction.

In the case of patient 13, the use of DWT instead of EMD considerably improved epileptic-seizure classification, i.e., an improvement from 0.775 to 0.820 using one EEG channel and from 0.777 to 0.849 using two. In general, both methods showed high accuracy when the EEG channels were selected using NSGA-based methods. The most-used classifiers when DWT was used for feature extraction were SVM and KNN for both NSGA-II and NSGA-III, as shown in a mesh plot of the most-used classifier for each patient (Figure 7). Specifically, for NSGA-II, RF was used an average of 20.5% ± 16.5 of the time for all patients, SVM 46.1% ± 23.5, NB 3.6% ± 3.8, and KNN 29.8% ± 23.1. When selecting the EEG channels using NSGA-III, the RF classifier was used an average of 22.1% ± 19.0 of the time, SVM 47.3% ± 24.5, NB 1.0% ± 1.4, and KNN 29.5% ± 23.3.

SVM was the most highly-used classifier in general, but RF and KNN were also highly used (Figure 7). These data also show that KNN is more highly used with DWT-based features than EMD-based features (see Figure 6). NB was the classifier with the lowest percentage of use for both approaches.

## 4. Discussion

We have presented a method for EEG channel selection for epileptic-seizure classification. Feature extraction was based on EMD or DWT. For each sub-band obtained, we then computed two energy and two fractal dimension features and the classification was performed automatically using four different classifiers to choose that with the highest accuracy.

The EEG channel selection method for epileptic-seizure classification proved to be robust. For example, the accuracy when using all EEG channels for patient 1 and DWT-based features was 0.97. The accuracy was even higher when using the EEG channels selected by NSGA-II or NSGA-III (1 or 2 channels): 0.98 for EMD and 1.00 for DWT.

As an example, the results obtained with the data of patient 12 show the highest accuracy using EMD to be 0.942 using six EEG channels selected by NSGA-III. The highest accuracy obtained using DWT-based features was 0.952 using four EEG channels. An important feature of the classification of the epileptic seizures of this patient is that most of the highest accuracies were obtained using the KNN classifier (see Figures 6, 7), i.e., an average of 73 and 84% when using EMD-based features, and an average of 96 and 98% using DWT-based features, for NSGA-II and NSGA-III respectively. Examination of the number of epileptic seizures described in the database (Goldberger et al., 2000) showed this patient to have had 38 and after segmentation (6-s segments), we obtained 234 instances of epileptic seizures and 234 for seizure-free periods. This amount of data is one of the highest of the patients used for this study [More details about the data are described by Moctezuma and Molinas (2019a)], however in the case of patient 15, which has a similar amount of data, the highest accuracies were obtained using SVM. Because of this, we cannot argue that this fact is because of the amount of data. Therefore, future work will also analyze more parameters related to the classifier (i.e., number of neighbors for KNN, and kernel as well as kernel parameters for SVM), how the accuracy is affected by the number of seizure periods/trials, and then we will figure out a possible relationship between the feature extraction method, the classifier and classifier's parameters, and more factors (Sample rate, wet or dry electrodes, EEG device, etc.) that can affect a solid conclusion.

As it is shown in Figures 6, 7, independently of the feature extraction method and if NSGA-II or NSGA-III is used for channel selection, SVM was the most highly-used classifier in general, but KNN was also highly used. These data also show that KNN is more highly used with DWT-based features than EMD-based features. NB was the classifier with the lowest percentage of use for both approaches, so, for our future steps, we will consider these findings and use that computational cost for testing other important parameters related to each classifier, instead of testing NB again.

In general, the results presented in this paper, have been shown that our approach is able to classify epileptic seizure and seizure-free periods with an accuracy up to 0.97 ± 0.05 in average, using only one EEG electrode. This result was obtained using DWT-based features, but if we use 2 or more channels, the accuracy increase to 0.98 and 0.99, specially when the EEG channels are selected by NSGA-III (see Table 5). In the state-of-the-art, there are some relevant works, where authors are presenting different methods for feature extraction and classification with the same dataset, under different experiment setups. Table 5 presents a general overview of that for analysis and comparison purposes.

**Table 5:** Comparison of relevant existing methods for epileptic seizures classification using the CHB-MIT Scalp EEG dataset presented in Shoeb (2009).

**References**	**Method**	**Subjects, channels**	**Evaluation**
Rafiuddin et al., 2011	Energy and coefficient of variation extracted from DWT, interquartile range, median absolute deviation from raw signal.	23, 23	0.80 of accuracy, using 80% for training.
Khan et al., 2012	Relative values of energy and a normalized coefficients of variation from DWT.	5, (23, 24, or 26)	0.91 of accuracy, using 80% for training.
Zabihi et al., 2015	Seven features from the intersection sequence of Poincaré section with phase space.	23, 23	0.93 and 0.94 of accuracies, using 25% and 50% for training, respectively.
Bhattacharyya and Pachori, 2017	Three features extracted from different oscillatory levels using multivariate extension of EWT.	23, 5	0.99 of accuracy, using 10-fold cross-validation.
Solaija et al., 2018	Signal curve length of the time-domain EEG signal and the mode powers of the dynamic mode decomposition.	12, 18	0.87 of sensitivity, using 50% for training.
Moctezuma and Molinas, 2019a	Teager and instantaneous energy, Higuchi and Petrosian fractal dimension, and DFA from 2 IMFs based on the EMD.	24,5	0.93 of accuracy in average, 10-fold cross-validation.
Proposed method using EMD-based features	Teager and instantaneous energy, Higuchi and Petrosian fractal dimension from 2 based on the EMD.	24, 1–3	0.93 ± 0.06, 0.95 ± 0.06, and 0.95 ± 0.05 of accuracies in average using 10-fold cross-validation for 1, 2, 3, and 4 channels selected by NSGA-II.
		24, 1–3 channels	0.93 ± 0.06, 0.94 ± 0.06, and 0.96 ± 0.04 of accuracies in average using 10-fold cross-validation for 1, 2, and 3 channels selected by NSGA-III.
Proposed method using DWT-based features	Teager and instantaneous energy, Higuchi and Petrosian fractal dimension from 4 decomposition levels of the DWT.	24, 1–3	0.97 ± 0.05, 0.97 ± 0.04, and 0.98 ± 0.02 in average using 10-fold cross-validation for 1, 2, and 3 channels selected by NSGA-II.
		24, 1–3	0.97 ± 0.05, 0.98 ± 0.03, and 0.99 ± 0.01 of accuracies in average using 10-fold cross-validation for 1, 2, and 3 channels selected by NSGA-III.

Table 5 shown the state-of-the-art and the classification accuracy of our approaches using EMD-based or DWT-based features, as well as NSGA-II or NSGA-III. It should be noted that our results are not directly comparable with previous works, since we are using a lowest amount of EEG channels, which were found by NSGA-based algorithms and we are using 24 subjects for the experiments, as well as different experimental setups. It should be noted that the average values presented in our results were obtained from Tables 1–4, which correspond to the results obtained in the Pareto-front for each subject in the dataset. Also when using 2 or 3 channels, the average accuracy is affected if for some subjects, the highest accuracies there were no obtained with that amount of EEG channels (See Tables 1–4), i.e., using features EMD-based the Pareto-front for NSGA-III is composed as: 0.992 of accuracy with 1 channel, and 1.00 of accuracy using 4 EEG channels, but there are no information about a combination with two or three channels for obtaining accuracies in the Pareto-front.

Most of the studies presented in Table 6, are based on invasive seizure EEG signals, which have better signal quality (Andrzejak et al., 2001). Therefore, their performance should be re-tested on non-invasive EEG signals for continuous monitoring. An interesting fact in the presented works is that SVM classifier is the most widely used, and it has exhibited the highest accuracies compared with other classifiers and neural networks, which is consistent with our own results.

**Table 6:** Comparison of some relevant existing methods for epileptic seizures classification using different datasets.

**References**	**Method**	**Subjects, channels**	**Evaluation**
Srinivasan et al., 2007	Features based on approximate entropy and classification using Elman and probabilistic neural networks.	5, 1	1.00 of accuracy.
Subasi and Gursoy, 2010	Five levels of decomposition using DWT and features using principal component analysis (PCA), independent component analysis (ICA), and LDA. The classification was using SVM.	5, 1	0.987, 0.995, and 1.00 of accuracies for features based on PCA, ICA and LDA, respectively.
Acharya et al., 2012	Entropies-Fuzzy Classifier with three classes, normal vs. pre-ictal vs. epileptic.	5, 1	0.981 of accuracy.
Sharma and Pachori, 2015	Features based on two-dimensional (2D) and three-dimensional (3D) PSRs of IMFs from EMD, and least-square SVM (LS-SVM) classifier.	5, 1	0.986 of accuracy.
Zhang et al., 2018	Using the TUH EEG corpus, they used 10-s segments with a sample rate of 250 Hz and they computed 24 features per channel. Six different classifiers were compared: SVM, NB, KNN, RF, gradient boosting and logistic regression.	43, 22	0.994 of accuracy using SVM.
Gupta and Pachori, 2019	Features based on Fourier-Bessel series expansion and classified using LS-SVM	5, 1	0.99 of accuracy in the best case.
Sharma et al., 2020	Third-order cumulant (ToC) and neural network with softmax classifier.	5, 1	1.00 of accuracy.
de la O Serna et al., 2020	Energy features from sub-bands extracted using the Taylor-Fourier filter bank and LS-SVM.	5, 1	0.948 of accuracy.

According to our results, NSGA-III is able to find the most relevant EEG channel combinations using DWT-based features for obtaining up to 0.99 of accuracy in average using only 3 channels, looking forward for improving the general performance of our proposal and for testing with more public dataset with epileptic seizures, we will propose new experiments considering more than two objective functions in the problem and verify if NSGA-III is still the best method for solving this problem (Deb and Jain, 2013; Jain and Deb, 2013). We show in our results that for some subjects the best accuracy can be reached using 1-3 channels and for others with more than 4 channels. For this reason, we propose as future work to test different methods trying to improve the channel selection process, and for decreasing the complexity. This can be by testing and comparing methods such as the one presented by Bhattacharyya and Pachori (2017), which selects a channel with the lowest SD and then four channels with the highest MI with the previously chosen channel.

The epileptic seizure classification using EEG signals is important for evaluating the state of the brain. The evolution of the signals by continuous monitoring (Panayiotopoulos and Koutroumanidis, 2005; Cho and Kim, 2019), will enable prediction with a low number of EEG channels and this will make it easier to use, allowing long-term monitoring using a possibly personalized portable EEG device. However, there are several challenges that must be addressed before implementation in real life. It is mainly because epilepsy can cause a variety of other neurological disorders (i.e., depression, anxiety, etc.) that it should be studied additionally to distinguish between an epileptic seizure and seizure-free. In that direction, our future efforts will also include the study of epilepsy-related disorders and how they can be recognized on EEG signals. A possible portable low-density EEG device will facilitate the monitoring in daily life, which will allow health care professionals more confident management of the seizures, through not only the service in a hospital or laboratory but also in conjunction with the recent ideas and progress in telehealth and telemedicine (Bingham and Patterson, 2007; Smith, 2016; Kissani et al., 2020).

From the results presented in this paper, we can figure out that EMD-based or DWT-based features can be useful for epileptic seizure classification, with this, a possible subject-tailored method can consider another gene in the chromosome for the optimization process and thus select the most useful method for detecting epileptic seizures for that subject. This will be tested in our future works, considering the findings here and also testing different chromosome representations for solving all the possible problems related to the parameters optimization at the same time.

The computational complexity of the method used for channel selection is *O*(*MN*^2^), in the best case. However, the study of the most relevant channels is important and it must be performed for analysis and as this work presented, to verify if the epileptic seizures can be detected using a few non-invasive EEG channels. The limitations of the methods used for feature extraction are related to the well-known problems of EMD, such as the selection of the best spline, the end effect, and the mode mixing problem (Huang et al., 1998; Rilling et al., 2003; Boutana et al., 2010). For DWT, the main problems are related to the parameters selection, such as the number of levels of decomposition and the mother function. Some of these limitations have been already considered in the literature or can be solved by using recent progress in code optimization (Lam et al., 2015; Dask Development Team, 2016; Blank and Deb, 2020), but other limitations are not yet well-established, and more research is necessary. Our future efforts for classification will be for testing and comparing shallow convolutional neural networks and Riemannian classifiers since they have been shown high accuracies for EEG signals classification (Kalunga et al., 2016; Schirrmeister et al., 2017; Lotte et al., 2018).

## Data Availability Statement

Publicly available datasets were analyzed in this study. This data can be found here: https://doi.org/10.13026/C2K01R.

## Ethics Statement

Ethical review and approval was not required for the study on human participants in accordance with the local legislation and institutional requirements. Written informed consent from the patients/participants OR patients/participants legal guardian/next of kin was not required to participate in this study in accordance with the national legislation and the institutional requirements.

## Author Contributions

LM devised the methods, performed the experiments, analyzed the results, and wrote the manuscript. MM devised the methods, discussed the results, and wrote the manuscript.

## Conflict of Interest

The authors declare that the research was conducted in the absence of any commercial or financial relationships that could be construed as a potential conflict of interest.
