# Supplementary material for: EEG Channel-Selection Method for Epileptic-Seizure Classification Based on Multi-Objective Optimization
Source: Front Neurosci. 2020 Jun 17;14:593. doi: 10.3389/fnins.2020.00593 (PMC7312219; doi:10.3389/fnins.2020.00593)
Supplement: Supplementary file 1 [file Data_Sheet_1.PDF]

frontiers

Supplementary Material

Table S1. Accuracy (Acc.), specificity (Spec.) and sensitivity (Sens.) obtained using EMD for feature extraction with NSGA-II and NSGA-III for EEG channel selection (Subjects 1-12).

Id	Method	No. channels											
		1			2			3			4		
		Acc	Spec.	Sens.	Acc	Spec.	Sens.	Acc	Spec.	Sens.	Acc	Spec.	Sens.
1	B-E	0.943	0.943	0.964	0.964	0.962	0.962	0.986	0.986	1.000	0.964	0.964	1.000
	NSGA-II	0.979	0.955	0.957	0.979	0.975	0.967	0.986	0.986	0.988	0.993	0.990	0.990
	NSGA-III	0.964	0.967	0.960	0.979	0.975	0.967						
2	B-E	0.815	0.810	0.825	0.899	0.899	0.909	0.921	0.921	0.935	0.921	0.921	0.935
	NSGA-II	0.866	0.866	0.876	0.921	0.921	0.921						
	NSGA-III	0.866	0.877	0.887									
3	B-E	0.796	0.790	0.792	0.888	0.888	0.888	0.912	0.912	0.915	0.920	0.921	0.931
	NSGA-II	0.911	0.911	0.910	0.943	0.943	0.952	0.958	0.958	0.966	0.975	0.975	0.977
	NSGA-III	0.876	0.876	0.888	0.927	0.927	0.930	0.951	0.940	0.945	0.975	0.973	0.977
4	B-E	0.832	0.832	0.832	0.940	0.940	0.940	0.948	0.948	0.948	0.977	0.977	1.000
	NSGA-II	0.914	0.914	0.914	0.946	0.946	0.946	0.955	0.955	0.955	0.977	0.977	0.985
	NSGA-III	0.897	0.897	0.897	0.955	0.955	0.955	0.963	0.972	1.000			
5	B-E	0.972	0.972	0.988	0.978	0.978	0.988	0.995	0.995	1.000	1.000	1.000	1.000
	NSGA-II	0.974	0.964	0.966	0.995	0.996	1.000	1.000	1.000	1.000			
	NSGA-III	0.970	0.970	0.983	0.995	0.995	1.000						
6	B-E	0.975	0.975	1.000	1.000	1.000	1.000	0.975	0.975	1.000	1.000	1.000	1.000
	NSGA-II	1.000	1.000	1.000	1.000	1.000	1.000						
	NSGA-III	1.000	1.000	1.000	1.000	1.000	1.000						
7	B-E	0.962	0.963	0.962	0.962	0.962	0.965	0.963	0.963	0.988	0.992	0.992	1.000
	NSGA-II	0.962	0.964	0.977	0.972	0.972	0.977	0.982	0.982	1.000	1.000	1.000	1.000
	NSGA-III	0.962	0.964	0.977	0.972	0.972	0.977				1.000	1.000	1.000
8	B-E	0.884	0.885	0.888	0.884	0.885	0.888	0.877	0.877	0.880	0.877	0.881	0.881
	NSGA-II	0.884	0.884	0.888	0.890	0.890	0.899	0.890	0.892	0.894	0.890	0.891	0.903
	NSGA-III	0.884	0.884	0.895	0.884	0.882	0.894						
9	B-E	1.000	1.000	1.000	1.000	1.000	1.000	1.000	1.000	1.000	1.000	1.000	1.000
	NSGA-II	1.000	1.000	1.000									
	NSGA-III	1.000	1.000	1.000									
10	B-E	0.993	0.993	0.993	0.993	0.993	1.000	0.993	0.993	1.000	1.000	1.000	1.000
	NSGA-II	0.993	0.993	1.000	1.000	1.000	1.000						
	NSGA-III	0.993	0.993	1.000	1.000	1.000	1.000						
11	B-E	0.996	0.996	0.996	0.996	0.996	1.000	0.996	0.996	1.000	0.992	0.992	1.000
	NSGA-II	0.996	0.996	1.000	0.996	0.996	1.000						
	NSGA-III	0.996	0.996	1.000	0.996	0.996	1.000						
12	B-E	0.899	0.899	0.909	0.892	0.892	0.905	0.918	0.918	0.928	0.911	0.921	0.925
	NSGA-II	0.899	0.805	0.758	0.908	0.884	0.885	0.919	0.883	0.865	0.928	0.876	0.899
	NSGA-III	0.899	0.834	0.764	0.912	0.889	0.775						

Table S2. Accuracy, specificity and sensitivity obtained using EMD for feature extraction with NSGA-II and NSGA-III for EEG channel selection (Subjects 13-24).

Id	Method	No. channels											
		1			2			3			4		
		Acc	Spec.	Sens.	Acc	Spec.	Sens.	Acc	Spec.	Sens.	Acc	Spec.	Sens.
13	B-E	0.775	0.776	0.788	0.777	0.777	0.781	0.775	0.775	0.779	0.806	0.807	0.816
	NSGA-II	0.775	0.775	0.782	0.777	0.7770	0.782	0.798	0.799	0.802	0.806	0.806	0.818
	NSGA-III	0.775	0.775	0.775	0.777	0.777	0.782						
14	B-E	0.925	0.925	0.925	0.933	0.933	0.933	0.942	0.942	0.942	0.942	0.942	0.942
	NSGA-II	0.933	0.933	0.933	0.967	0.967	1.000	0.983	0.983	1.000	0.983	0.983	1.000
	NSGA-III	0.933	0.933	0.942	0.942	0.942	0.944	0.983	0.983	0.992			
15	B-E	0.971	0.971	0.971	0.969	0.972	0.974	0.978	0.981	0.982	0.981	0.982	1.000
	NSGA-II	0.981	0.981	0.982	0.981	0.981	0.982	0.988	0.988	0.992	0.988	0.988	1.000
	NSGA-III	0.981	0.981	0.981	0.985	0.985	0.985	0.988	0.988	0.991			
16	B-E	0.900	0.900	0.901	0.900	0.901	0.901	0.900	0.900	0.902	0.900	0.900	0.911
	NSGA-II	0.900	0.900	0.901	0.900	0.900	0.910						
	NSGA-III	0.900	0.900	0.901	0.900	0.900	0.910						
17	B-E	0.940	0.940	0.940	0.980	0.982	0.985	0.980	0.980	1.000	0.990	0.990	1.000
	NSGA-II	0.980	0.982	0.992	0.990	0.990	1.000	1.000	1.000	1.000			
	NSGA-III	0.980	0.982	0.992				1.000	1.000	1.000			
18	B-E	0.790	0.790	0.790	0.852	0.852	0.852	0.832	0.832	0.832	0.862	0.862	0.862
	NSGA-II	0.803	0.803	0.803	0.852	0.852	0.852	0.870	0.870	0.870	0.900	0.900	0.922
	NSGA-III	0.783	0.784	0.786	0.852	0.852	0.852	0.862	0.862	0.862	0.880	0.880	0.880
19	B-E	0.913	0.913	0.913	0.908	0.908	0.908	0.925	0.925	0.925	0.925	0.925	0.925
	NSGA-II	0.921	0.921	0.921	0.946	0.946	0.946	0.950	0.950	0.952	0.963	0.963	0.972
	NSGA-III	0.913	0.913	0.913	0.975	0.974	0.976						
20	B-E	0.948	0.948	0.948	0.970	0.970	0.970	0.957	0.957	0.957	0.957	0.957	0.957
	NSGA-II	0.980	0.982	0.987				0.990	0.990	1.000			
	NSGA-III	0.980	0.982	0.987				0.990	0.990	1.000			
21	B-E	0.879	0.879	0.879	0.933	0.933	0.933	0.888	0.888	0.888	0.888	0.888	0.888
	NSGA-II	0.888	0.888	0.888	0.950	0.950	0.950	0.954	0.954	0.954	0.967	0.967	0.976
	NSGA-III	0.888	0.888	0.888	0.942	0.942	0.942	0.954	0.954	0.954	0.983	0.983	1.000
22	B-E	0.971	0.971	0.971	0.971	0.971	0.971	0.983	0.989	0.992	0.983	0.985	0.995
	NSGA-II	0.983	0.983	0.983				0.983	0.985	0.988			
	NSGA-III	0.983	0.983	0.983									
23	B-E	0.938	0.938	0.938	0.940	0.940	0.940	0.938	0.938	0.938	0.955	0.955	0.956
	NSGA-II	0.938	0.938	0.938	0.948	0.948	0.948	0.962	0.962	0.966			
	NSGA-III	0.938	0.938	0.938	0.946	0.948	0.950						
24	B-E	0.975	0.975	0.975	0.975	0.975	0.975	0.992	0.993	1.000	0.992	0.993	1.000
	NSGA-II	0.975	0.975	0.975	0.992	0.992	1.000	0.992	0.992	0.997	1.000	1.000	1.000
	NSGA-III	0.992	0.992	1.000							1.000	1.000	1.000

Table S3. Accuracy, specificity and sensitivity obtained using DWT for feature extraction with NSGA-II and NSGA-III for EEG channel selection (Subjects 1-12).

**Id**	**Method**	**No. channels**											
		**1**			**2**			**3**			**4**		
		**Acc**	**Spec.**	**Sens.**	**Acc**	**Spec.**	**Sens.**	**Acc**	**Spec.**	**Sens.**	**Acc**	**Spec.**	**Sens.**
1	B-E	0.950	0.950	0.950	0.993	0.993	1.000	0.993	0.993	1.000	0.993	0.993	1.000
	NSGA-II	0.986	0.979	0.986	1.000	1.000	1.000						
	NSGA-III	0.986	0.979	0.986				1.000	1.000	1.000			
2	B-E	0.983	0.983	0.985	0.992	0.992	1.000	0.992	0.992	1.000	1.000	1.000	1.000
	NSGA-II	0.992	0.992	0.991	0.992	0.992	0.991	1.000	1.000	1.000			
	NSGA-III	0.992	0.992	0.991	0.992	0.992	0.991				1.000	1.000	1.000
3	B-E	0.983	0.983	0.983	0.985	0.985	0.985	0.992	0.992	1.000	1.000	1.000	1.000
	NSGA-II	0.983	0.982	0.975	0.992	0.992	0.991	1.000	1.000	1.000			
	NSGA-III	0.983	0.983	0.978				1.000	1.000	1.000			
4	B-E	0.952	0.952	0.951	0.966	0.966	0.966	0.975	0.975	0.972	0.983	0.983	0.981
	NSGA-II	1.00	1.000	1.000									
	NSGA-III	1.00	1.000	1.000									
5	B-E	0.995	0.995	1.000	1.000	1.000	1.000	1.000	1.000	1.000	1.000	1.000	1.000
	NSGA-II	1.000	1.000	1.000									
	NSGA-III	1.000	1.000	1.000									
6	B-E	0.975	0.975	0.975	0.950	0.950	0.950	0.950	0.950	0.950	0.950	0.950	0.950
	NSGA-II	0.975	0.975	0.973	0.975	0.975	0.974	0.975	0.975	0.970			
	NSGA-III	0.975	0.975	0.973	0.975	0.975	0.974						
7	B-E	0.962	0.962	0.962	0.972	0.972	0.972	0.980	0.980	0.982	0.980	0.981	0.983
	NSGA-II	0.980	0.978	0.973	0.982	0.980	0.982	1.000	1.000	1.000			
	NSGA-III	0.980	0.980	0.975				1.000	1.000	1.000			
8	B-E	0.914	0.914	0.915	0.903	0.903	0.903	0.917	0.917	0.917	0.904	0.904	0.911
	NSGA-II	0.917	0.910	0.911	0.917	0.910	0.910						
	NSGA-III	0.971	0.910	0.911				0.917	0.910	0.910			
9	B-E	1.000	1.000	1.000	1.000	1.000	1.000	1.000	1.000	1.000	1.000	1.000	1.000
	NSGA-II	1.000	1.000	1.000	1.000	1.000	1.000						
	NSGA-III	1.000	1.000	1.000									
10	B-E	1.000	1.000	1.000	1.000	1.000	1.000	1.000	1.000	1.000	1.000	1.000	1.000
	NSGA-II	1.000	1.000	1.000									
	NSGA-III	1.000	1.000	1.000	1.000	1.000	1.000						
11	B-E	1.000	1.000	1.000	1.000	1.000	1.000	1.000	1.000	1.000	1.000	1.000	1.000
	NSGA-II	1.000	1.000	1.000									
	NSGA-III	1.000	1.000	1.000									
12	B-E	0.899	0.899	0.919	0.932	0.933	0.933	0.942	0.942	0.943	0.942	0.942	0.950
	NSGA-II	0.911	0.914	0.905	0.948	0.941	0.925	0.948	0.948	0.945	0.952	0.950	0.945
	NSGA-III	0.911	0.914	0.905									

Table S4. Accuracy, specificity and sensitivity obtained using DWT for feature extraction with NSGA-II and NSGA-III for EEG channel selection (Subjects 13-24).

Id	Method	No. channels											
		1			2			3			4		
		**Acc**	**Spec.**	**Sens.**	**Acc**	**Spec.**	**Sens.**	**Acc**	**Spec.**	**Sens.**	**Acc**	**Spec.**	**Sens.**
13	B-E	0.822	0.822	0.824	0.827	0.827	0.827	0.793	0.793	0.802	0.827	0.827	0.833
	NSGA-II	0.820	0.822	0.830	0.849	0.849	0.851				0.855	0.855	0.857
	NSGA-III	0.820	0.822	0.830							0.850	0.850	0.852
14	B-E	0.950	0.950	0.952	0.967	0.967	0.968	0.983	0.983	0.992	0.983	0.984	0.992
	NSGA-II	0.967	0.967	0.968	0.983	0.983	0.983	0.995	0.995	1.000			
	NSGA-III	0.967	0.967	0.968	0.983	0.983	0.983				1.000	1.000	1.000
15	B-E	0.978	0.978	0.980	0.985	0.985	0.988	0.981	0.981	0.982	0.986	0.986	0.989
	NSGA-II	0.978	0.978	0.982	0.994	0.994	0.998	1.000	1.000	1.000			
	NSGA-III	0.978	0.978	0.982	0.994	0.994	0.998	0.998	0.998	1.000			
16	B-E	0.800	0.800	0.810	1.000	1.000	1.000	1.000	1.000	1.000	1.000	1.000	1.000
	NSGA-II	1.000	1.000	1.000									
	NSGA-III	1.000	1.000	1.000									
17	B-E	0.930	0.931	0.935	1.000	1.000	1.000	1.000	1.000	1.000	1.000	1.000	1.000
	NSGA-II	1.000	1.000	1.000									
	NSGA-III	1.000	1.000	1.000									
18	B-E	0.862	0.862	0.866	0.862	0.862	0.866	0.912	0.912	0.931	0.922	0.925	0.931
	NSGA-II	0.890	0.890	0.890	0.913	0.913	0.913	0.950	0.950	0.950			
	NSGA-III	0.862	0.862	0.862	0.913	0.913	0.913						
19	B-E	0.987	0.987	0.987	1.000	1.000	1.000	0.987	0.991	0.992	1.000	1.000	1.000
	NSGA-II	0.988	0.988	0.993	1.000	1.000	1.000						
	NSGA-III	0.988	0.988	0.993	1.000	1.000	1.000						
20	B-E	1.000	1.000	1.000	1.000	1.000	1.000	1.000	1.000	1.000	1.000	1.000	1.000
	NSGA-II	1.000	1.000	1.000									
	NSGA-III	1.000	1.000	1.000									
21	B-E	0.921	0.921	0.922	0.950	0.953	0.953	0.938	0.939	0.945	0.967	0.967	0.981
	NSGA-II	0.925	0.925	0.928	0.950	0.950	0.960	0.971	0.971	0.972	0.983	0.983	0.985
	NSGA-III	0.933	0.933	0.934	0.950	0.950	0.955						
22	B-E	0.983	0.983	0.984	0.983	0.985	0.985	0.983	0.985	0.985	0.983	0.985	0.985
	NSGA-II	0.995	0.995	1.000	0.998	0.999	1.000	1.000	1.000	1.000			
	NSGA-III	0.995	0.995	1.000	0.995	0.999	1.000						
23	B-E	0.938	0.938	0.942	0.946	0.946	0.946	0.953	0.953	0.955	0.961	0.961	0.968
	NSGA-II	0.939	0.939	0.940	0.961	0.961	0.968	0.969	0.969	0.973	0.970	0.972	0.972
	NSGA-III	0.939	0.939	0.940	0.961	0.964	0.971						
24	B-E	0.975	0.975	0.976	0.975	0.976	0.976	0.975	0.976	0.976	0.975	0.976	0.976
	NSGA-II	0.985	0.986	0.990	0.992	0.993	1.000	1.000	1.000	1.000			
	NSGA-III	0.985	0.986	0.990	0.988	0.988	0.989				1.000	1.000	1.000
